# INSIGHT responsive parenting intervention and infant feeding practices: randomized clinical trial

**DOI:** 10.1186/s12966-018-0700-6

**Published:** 2018-07-09

**Authors:** Jennifer S. Savage, Emily E. Hohman, Michele E. Marini, Amy Shelly, Ian M. Paul, Leann L. Birch

**Affiliations:** 1 Center for Childhood Obesity Research, 129 Noll Laboratory, The Pennsylvania State University, University Park, PA 16802 USA; 2 Department of Nutritional Sciences, The Pennsylvania State University, University Park, PA USA; 3 Pediatrics and Public Health Sciences, Penn State College of Medicine, Hershey, PA USA; 4 Department of Foods and Nutrition, University of Georgia, Athens, GA USA

**Keywords:** Obesity prevention, Infancy, Responsive parenting, Feeding practices and styles, Bottle-feeding and diet

## Abstract

**Background:**

What, when, how, how much, and how often infants are fed have been associated with childhood obesity risk. The objective of this secondary analysis was to examine the effect of a responsive parenting (RP) intervention designed for obesity prevention on parents’ infant feeding practices in the first year after birth.

**Methods:**

Primiparous mother-newborn dyads were randomized to the Intervention Nurses Start Infants Growing on Healthy Trajectories (INSIGHT) Study RP intervention or child safety control. Research nurses delivered intervention content at home at infant age 3–4, 16, 28, and 40 weeks, and at a research center at 1 year. RP feeding guidance advised feeding that was contingent (i.e., feed in response to hunger and satiety signs, alternatives to using food to soothe), and developmentally appropriate (i.e., delaying introduction of solids, age-appropriate portion sizes). Infant feeding practices (i.e., bottle use, introduction of solids, food to soothe) were assessed by phone interviews and online surveys and dietary intake was assessed using a food frequency questionnaire.

**Results:**

RP mothers were more likely to use of structure-based feeding practices including limit-setting (*p* < 0.05) and consistent feeding routines (*p* < 0.01) at age 1 year. RP group mothers were less likely to use non-responsive feeding practices such as pressuring their infant to finish the bottle/food (*p* < 0.001), and using food to soothe (*p* < 0.01), propping the bottle (*p* < 0.05) assessed between 4 and 8 months, and putting baby to bed with a bottle at age 1 year (*p* < 0.05). Few differences were seen between groups in *what* specific foods or food groups infants were fed.

**Conclusions:**

Anticipatory guidance on RP in feeding can prevent the use of food to soothe and promote use of more sensitive, structure-based feeding which could reduce obesity risk by affecting how and when infants are fed during the first year.

**Trial registration:**

The Intervention Nurses Start Infants Growing on Healthy Trajectories (INSIGHT) Study. www.clinicaltrials.gov . NCT01167270. Registered 21 July 2010.

**Electronic supplementary material:**

The online version of this article (10.1186/s12966-018-0700-6) contains supplementary material, which is available to authorized users.

## Background

Epidemiological and laboratory findings point to early modifiable risk factors to prevent rapid weight gain and overweight [1, 2] including what, when, and how much an infant is fed [3]. What infants are fed in the first 24 months shapes their growth and subsequent dietary patterns [4], but how infants are fed can also influence early growth and development. There is evidence that US infants are often overfed, exceeding their estimated energy requirements [4].

Using a larger volume bottle to feed (i.e., > 6 oz) is associated with greater formula intake and rapid weight gain [5–7]. Controlling feeding practices, including pressuring the infant to finish the bottle and using feeding as the default response to any infant distress, reduced opportunities for infants self-regulation and can promote excessive weight gain [8]. In sum, how parents shape children’s early experiences with food and eating, by teaching the child food rules and expectations, impacts child taste preferences, food choice, appetite regulation, dietary intake and obesity risk [9–13].

The theoretical framework for the *I*ntervention *N*urses *S*tart *I*nfants *G*rowing on *H*ealthy *T*rajectories (*INSIGHT*) trial is based on responsive parenting (RP). RP is defined as responding to the infant promptly, contingently, in ways that are developmentally appropriate [14]. Previous findings indicate that RP is associated with a range of positive child outcomes [15–18]. INSIGHT’s central hypothesis was that responsive infant feeding guidance, focused on how to recognize and respond to infant hunger and fullness signs, could promote infant self-regulation, while also reducing the use of less responsive feeding practices (e.g., using feeding as the default response to any infant distress or promoting bottle emptying) would reduce risk for overeating and overweight among first-born infants [19]. This is based in part on evidence that feeding in response to infant hunger and fullness cues supports the development of appetite regulation (i.e., eating in response to hunger cues and not eating beyond satiation) [20, 21]. In contrast, use of non-responsive, coercive and controlling feeding practices, characterized by parents providing few opportunities for children to make choices about food and eating and to develop self-regulatory skills (e.g., feeding to quiet a distressed infant who is not hungry), can promote overfeeding, rapid weight gain and obesity risk [22–25].

The INSIGHT curriculum included guidance on feeding, including how to recognize and distinguish hunger from other distress, taught parents to accurately identify fullness signs to prevent overfeeding, instructed parents on a variety of alternative soothing strategies to manage infant crying (e.g., swaddling, non-nutritive sucking) to prevent the use of food as a first response to crying, and provided parents active learning opportunities to practice these skills with a trained nurse. Infants randomized to the INSIGHT RP intervention had slower weight gain during the first 6 months after birth and a reduced prevalence of overweight at 1 year [26]. The aim of this secondary analysis was to assess the proximal effects of the RP INSIGHT intervention on parent feeding styles and behaviors during the first year after birth. The hypothesis for this analysis was that responsive feeding practices would be more prevalent among mothers randomized to the RP group relative to control.

## Methods

### Subjects and study design

Mothers and newborns were recruited into the INSIGHT study from one maternity ward in central Pennsylvania between January 2012 and March 2014. Mothers were eligible for the study if they were primiparous, English-speaking, and ≥ 20 years of age, and if their newborns were full-term (≥37 weeks gestation), singleton, and weighed ≥2500 g at birth. Using a computer-generated algorithm, mother-infant dyads were randomized 2 weeks after delivery to either an RP intervention or a safety control group, stratified on birth weight for gestational age (<50th percentile or ≥ 50th percentile) and intended milk-feeding type. A baseline survey was administered to mothers electronically. Further details on study design, recruitment/eligibility, and a CONSORT diagram have been previously published [19, 26].

Following randomization, preliminary intervention materials were mailed to all participants. As described previously [19], research nurses were trained in administering both the RP and safety control interventions at home visits conducted at child age 3–4, 16, 28, and 40 weeks, and research center visit at 52 weeks. 291 mother-infant dyads were randomized. 279 mother-infant dyads completed the first home visit at 3–4 weeks and are considered the study cohort for all outcomes and analyses as specified prior to study initiation. At 28 and 52 weeks, 269 (96.4%) and 253 (90.7%) dyads remained in the study, and there was no significant difference in attrition by study group. This study was approved by the Human Subjects Protection Office of the Penn State College of Medicine’s Human Protection Office and registered at http://www.clinicaltrials.gov prior to participant enrollment. Mothers provided written consent for their and their infant’s participation.

### RP intervention feeding-related components

The INSIGHT intervention focused on RP in four domains of infant behavior: drowsy, sleeping, fussy, and alert and calm. A more detailed description of the RP and control curricula has been previously published [19]. Feeding guidance during infancy included (1) recognizing and responding appropriately to infant hunger and satiety cues; (2) age-appropriate, bottle-feeding practices (i.e., bottle size, nipple flow, transition off bottle); (3) delaying introduction to solids until 4–6 months; (4) promoting acceptance, liking, and intake of developmentally appropriate foods such as vegetables through repeated exposure; (5) serving age appropriate portions of healthy foods; and (6) using structure-based, non-controlling feeding practices that allow the infant to affect intake through shared control of the initiation and termination of feedings. Major themes of the feeding curriculum at each time point are listed in Table 1. RP guidance for fussy infants in the first year involved teaching parents to recognize hunger and distinguish hunger from other infant distress, and to how to use alternatives to feeding (e.g. swaddling, swinging, offering pacifier) to soothe/calm a fussy infant to promote healthy self-regulation development, including learning to self-soothe. The control group received an intervention that was similar in intensity, but focused on home safety. Feeding-related messaging in the safety curriculum was focused on food safety and choking prevention.

**Table 1:** Feeding messages in the responsive parenting intervention curriculum

Intervention component	Child age in weeks			
	3–4	16	28	40
What to feed				
Only breastmilk or formula for first 4–6 months	x			
No cereal in bottle	x	x		
Avoid/limit fruit juice	x	x	x	x
Portion sizes for complementary foods		x	x	x
Fruits and vegetables		x	x	x
Foods to limit		x	x	x
Water			x	x
Snacking				x
Cow’s milk				x
When to feed				
Introducing solids at 4–6 months	x	x		
Introducing cup at 6–9 months			x	x
Weaned from bottle by 1 year			x	x
Introducing a spoon				x
How to feed				
Bottle size & nipple type	x	x		
Hunger and fullness cues	x	x	x	
Do not put to bed with bottle/cup	x	x	x	x
Alternatives to using food to soothe	x	x	x	x
Do not pressure child to eat	x	x	x	x
Repeated exposure to new foods		x	x	x
Modeling positive eating behavior		x	x	x
Shared responsibility of feeding		x	x	x
Mealtime routines			x	x
Family meals				x
Eating away from home				x
Do not use food to reward/control behavior				x
Purchasing and access to unhealthy foods				x

### Measures

Phone interviews (conducted by study personnel other than the intervention nurses) and surveys were used to collect data. Online surveys, or paper surveys for those lacking Internet connectivity (*n* = 20), were sent to participants 2–3 weeks prior to each visit. Data were collected and managed using REDCap [27]. A detailed list of measures has been published [19]. Demographic information was collected from participants at enrollment (e.g., parent and child race/ethnicity, marital status). Maternal age, pre-pregnancy weight, gestational weight gain, and infant gestational age, sex, and birth weight and length were abstracted from medical charts.

#### Bottle-feeding and breastfeeding practices

Information on developmentally-relevant infant feeding practices was obtained at infant age 8, 20, 32, and 52 weeks. Mothers were asked about bottle-feeding practices (i.e. bottle and nipple size, adding cereal to the bottle, putting child to bed with a bottle or sippy cup), formula and breastfeeding practices (what they were currently feeding, cessation of breastfeeding). Responsiveness to fullness signals was assessed by asking mothers how frequently they tried to get the infant to finish the bottle.

#### Introduction of complementary foods

Mothers reported on the introduction of solids (when and what foods were introduced first).

#### Feeding beliefs and behaviors

At 28 weeks, mothers completed the Infant Feeding Styles Questionnaire (IFSQ) designed to assess feeding beliefs and behaviors among parents of infants in 5 feeding style domains: laissez-faire, pressuring, restrictive, responsive, and indulgent [28]. In this analysis, results for the pressuring (pressure to finish, α = 0.81; pressure with cereal, α = 0.83; pressure to soothe, α = 0.79) restrictive (restrictive amount, α = 0.74; restrictive diet quality, α = 0.69), and responsive satiety (α = 0.77) feeding styles are presented. The laissez-faire attention, laissez-faire diet quality, and responsive attention scales demonstrated poorer reliability (α = 0.62, 0.53, and 0.62) in our sample, while the indulgent subscale questions were not relevant at the 28 week time point, and thus these scales were not further analyzed.

At 1 year, the Structure and Control in Parent Feeding (SCPF) questionnaire was used to assess controlling feeding practices: Pressure to Eat (α = 0.78) and Restriction (α = 0.68) [29]. In addition, this scale assesses responsive feeding practices that may promote self-regulation, including Limiting Exposure to Unhealthy Foods (α = 0.72) and Consistent Feeding Routines (α = 0.76).

#### Feeding to soothe

Use of feeding to soothe a fussy infant was assessed at 8, 16, 32, and 44 weeks using items from the Baby’s Basic Needs questionnaire [24]. This scale was modified to include contexts and situations when a mother could use feeding to soothe. An unweighted least squares exploratory factor analysis (EFA) was completed for the 21-item survey, using an equamax rotation (see Additional file 1). Items were iteratively removed based on loadings that were < 0.4 producing a factor structure with 2 eigenvalues > 1. Restricting the EFA to 2 factors gave factor loadings with simple structure, with two feeding-to-soothe factors: contextual (6 items, α = 0.79) and emotional (6 items; α = 0.90). Higher contextual feeding-to-soothe scores indicate greater use of feeding to soothe or quiet a distressed child in a variety of contexts (i.e., in a doctor’s waiting room, in the car, before bed), without regard for whether hunger was the source of infant distress. Higher emotional feeding-to-soothe scores indicate greater use of feeding to soothe in response to either the infant’s distress, or maternal stress, frustration, or anger. Mothers also rated the effectiveness of using feeding to soothe their infant on a scale of 1 (does not work) to 4 (works all of the time).

#### Food intake

Mothers reported the frequency of serving 24 beverages and 104 solid food items during the past week at 3–4, 16, 28, and 40 weeks to assess frequency of breast and formula feeding and complementary food intake on a food frequency questionnaire. Predominant breastfeeding was defined if ≥80% of milk feedings were breastmilk, [30] either at the breast or by bottle [25, 31]. Frequencies for other food and beverage items were recoded to times per day, and total times consumed per day for food groups (fruit – 18 items, vegetables – 22 items, sweets – 12 items, salty snacks − 5 items, sugar-sweetened beverages – 7 items, and fried foods − 3 items) were calculated. The percent of participants consuming each food group at least once in the past week and at least once per day was also calculated.

### Statistical analysis

Data were analyzed using SAS 9.4 (SAS Institute, Cary, NC). The effect of study group on categorical outcomes was assessed using logistic regression, and effect on continuous variables was using ANOVA. Odds ratios and Cohen’s D were calculated as indicators of effect size for logistic regression and ANOVA analyses, respectively. All analyses controlled for the randomization stratification variables. Effects of predominant milk-feeding type (breast vs. formula) and interactions between study group and milk-feeding type were also assessed for dietary outcomes. Significance was defined as *p* < 0.05.

## Results

Participating mothers were primiparous, predominantly white, non-Hispanic, married, and college educated with about 50% reporting annual household incomes above $75,000 (Table 2). There were no significant differences in baseline characteristics between the RP and control groups. At the 3–4 week home visit, 60.9% of infants were predominantly breastfed, and this dropped to 47.0% at 16 weeks, 37.0% at 28 weeks, and 32.7% at 40 weeks. There were no significant study group differences in the proportion of infants who were predominantly breastfed at any time point.

**Table 2:** Participant demographics (*n* = 279)

	RP (*n* = 140)	Control (*n* = 139)
Infant		
Male sex, N (%)	75 (53.6)	69 (49.6)
Gestational age (weeks), mean (SD)	39.6 (1.2)	39.5 (1.1)
Birth weight (kg), mean (SD)	3.40 (0.43)	3.46 (0.43)
Birth length (cm), mean (SD)	50.9 (2.4)	51.0 (2.1)
Mother		
Age (years), mean (SD)	28.7 (4.6)	28.7 (4.9)
Pre-pregnancy BMI, mean (SD)	25.5 (5.0)	25.3 (5.6)
Gestational weight gain (kg), mean (SD)	15.6 (6.4)	15.0 (6.0)
Diabetes during pregnancy, *N* (%)	6 (4.3)	13 (9.4)
Smoked during pregnancy, *N* (%)	12 (8.6)	9 (6.5)
Race, *N* (%)		
Black	10 (7.1)	7 (5.0)
White	122 (87.1)	127 (91.4)
Native Hawaiian or Pacific Islander	1 (0.7)	0 (0)
Asian	5 (3.6)	4 (2.9)
Other	2 (1.4)	1 (0.7)
Ethnicity, *N* (%)		
Hispanic/Latino	12 (8.6)	7 (5.0)
Marital status, *N* (%)		
Married	102 (72.9)	108 (77.7)
Not married, living with partner	25 (17.9)	19 (13.7)
Single	12 (8.6)	12 (8.6)
Divorced/separated	1 (0.7)	0 (0)
Annual household income, *N* (%)		
< $10,000	6 (4.3)	5 (3.6)
$10,000–$24,999	10 (7.1)	10 (7.2)
$25,000–$49,999	5 (3.6)	23 (16.6)
$50,000–$74,999	46 (32.9)	26 (18.7)
$75,000–$99,999	32 (22.9)	23 (16.6)
$100,000 or more	32 (22.9)	43 (30.9)
Do not know or refuse to answer	9 (6.4)	9 (6.4)
Education, *N* (%)		
HS graduate or less	16 (11.4)	16 (11.5)
Some college	37 (26.4)	36 (25.9)
College graduate	48 (34.3)	52 (37.4)
Graduate degree +	39 (27.9)	35 (25.2)

### Bottle-feeding practices

As shown in Table 3, few participating mothers reported they were adding cereal to their child’s bottle at 8 weeks, but at 20 weeks, fewer mothers in the RP group were adding cereal to the bottle (8.4% vs 20.0% in the control group). At 1 year, children in the RP group were more likely to have transitioned their infant off the bottle (37.6%) than children in the control group (21.1%). The majority of mothers in both groups reported using a slow-flow nipple at 8 weeks, but at 20 weeks, a greater percentage of mothers in the RP group were still using a slow-flow nipple compared to those in the control group (83/117 (70.9%) vs 61/105 (58.1%), *p* = 0.050) (see Table 3). There were no differences between groups in the percentage of mothers using a larger bottle size (> 8 oz) at 8, 20 or 32 weeks. Fewer RP mothers reported propping the bottle at 28 weeks (10.1%) compared to control (19.7%). At age 1 year, more RP mothers were using a sippy cup than control. At 8, 20, and 32 weeks, the percentage of mothers who reported “ever” putting their child to bed with a bottle was relatively low and did not differ between groups, but at 52 weeks, fewer mothers in the RP group reported this behavior (10.4% vs 20.3% in the control group). Mothers in the RP group also fed their infant fewer times at night (7 pm-7 am) than control at 20 and 32 weeks, but not 8 weeks.

**Table 3:** Effect of RP intervention on bottle feeding

	RP (*n* = 140)	Control (*n* = 139)	*p*-value^a^	Odds ratio (CI) or Cohen’s D
Currently adding cereal to bottle (denominator = current bottle users)				
8 weeks	6/114 (5.3%)	2/112 (1.8%)	0.18	3.1 (0.6–15.6)
20 weeks	10/119 (8.4%)	22/110 (20.0%)	0.01	0.4 (0.2–0.8)
32 weeks	11/108 (10.2%)	15/107 (14.0%)	0.38	0.7 (0.3–1.6)
Uses slow flow nipple (denominator = current bottle users)				
8 weeks	101/114 (88.6%)	86/107 (80.4%)	0.10	1.9 (0.9–4.0)
20 weeks	83/117 (70.9%)	61/105 (58.1%)	0.05	1.8 (1.0–3.2)
Use bottle ≥8 oz. (denominator = current bottle users)				
8 weeks	10/111 (9.0%)	15/106 (14.1%)	0.26	0.6 (0.3–1.4)
20 weeks	37/117 (31.6%)	43/105 (40.9%)	0.17	0.7 (0.4–1.2)
32 weeks	49/103 (47.6%)	57/102 (55.9%)	0.28	0.7 (0.4–1.3)
Prop/Propped the bottle (denominator = all available)				
16 weeks	17/122 (13.9%)	22/117 (18.6%)	0.30	0.7 (0.3–1.4)
28 weeks	13/129 (10.1%)	24/122 (19.7%)	0.03	0.4 (0.2–0.9)
Using bottle (denominator = all available)				
52 weeks	78/125 (62.4%)	97/123 (78.9%)	0.005	0.4 (0.3–0.8)
Using sippy cup (denominator = all available)				
32 weeks	54/128 (42.2%)	42/129 (32.6%)	0.11	1.5 (0.9–2.5)
52 weeks	124/125 (99.2%)	113/125 (90.4%)	0.01	13.2 (1.7–104)
Using regular cup (denominator = all available)				
32 weeks	9/127 (7.1%)	10/131 (7.6%)	0.85	0.9 (0.4–2.3)
52 weeks	14/125 (11.2%)	9/125 (7.2%)	0.29	1.6 (0.7–3.9)
Puts child to bed with bottle/sippy cup (denominator = current bottle or sippy cup users)				
8 weeks	0/114 (0%)	3/108 (2.8%)	0.95	NA
20 weeks	2/118 (1.7%)	3/106 (2.8%)	0.56	0.6 (0.1–3.6)
32 weeks	6/114 (5.3%)	9/113 (7.8%)	0.42	0.6 (0.2–1.9)
52 weeks	13/125 (10.4%)	25/122 (20.5%)	0.03	0.5 (0.2–0.9)
Night-time (7 pm-7 am) feedings – mean (SD)				
8 weeks	3.1 (1.2), *n* = 127	3.3 (1.3), *n* = 130	0.20	0.16
20 weeks	1.8 (1.2), *n* = 123	2.2 (1.3), *n* = 123	0.03	0.32
32 weeks	1.3 (1.1), *n* = 122	1.7 (1.4), *n* = 121	0.01	0.32

^a^*p* for study group difference, ANOVA or logistic regression adjusted for stratification variables

### Transition to complementary foods

In both the RP and control groups, the most common first food was infant cereal (71/124 (57.3%) in the RP group and 72/124 (58.1%) in control introduced cereal first), but among those who offered a different first food, mothers in the RP group were more likely to introduce vegetables first (50/124 (40.3%)) compared to control (31/124 (25.0%)). Mothers in the RP group reported introducing solids to their infant later than those in the control group (mean 22.0 ± SD 3.9 weeks vs. 21.0 ± 4.2 weeks, difference 1.0 (95% CI: 0.04–1.98) weeks, *p* = 0.04); however, the majority of mothers in both groups introduced solids between 4 and 6 months (17–26 weeks) (RP = 113/140 (80.7%), control = 117/139 (84.2%)), consistent with current guidance. Introduction before or after 6 months did not differ significantly between groups. Infants who were predominantly breastfed at 16 weeks were introduced to solids later than formula fed infants (22.6 ± 3.6 weeks vs. 20.4 ± 4.2 weeks, *p* = 0.003), but there was no interaction between study group and predominant milk-feeding type.

### Use of pressure, restriction, and structure in infant feeding

As shown in Table 4, at 8, 20, and 32 weeks, mothers in the parenting group were less likely to report that they encouraged their child to finish the bottle if their infant stopped drinking before the milk was gone. In addition, more mothers in the control group reported “ever” trying to get their child to finish breastmilk/formula or food at 28 weeks, but not at 16 weeks, compared to RP mothers. At 28 weeks, mothers in the RP group had lower scores on all three pressure subscales of the IFSQ (pressure to finish, pressure to soothe, and pressure with cereal) than mothers in the control group (Table 4); there was no difference on restriction or responsive satiety. At 52 weeks, mothers in the RP group reported lower use of pressure to eat and greater use of the two structure-based feeding scales, limiting exposure to unhealthy foods, and consistent feeding routines.

**Table 4:** Effect of RP intervention on maternal pressure, restriction, and structure in feeding

	RP (*n* = 140)	Control (*n* = 139)	*p*-value^a^	Odds ratio (CI) or Cohen’s D
Pressuring feeding				
Child is encouraged to finish a bottle if baby stops drinking before the milk is all gone (≥sometimes, denominator = current bottle users)				
8 weeks	38/114 (33.3%)	56/108 (51.4%)	0.007	0.5 (0.3–0.8)
20 weeks	41/117 (35.0%)	53/105 (50.5%)	0.02	0.5 (0.3–0.9)
32 weeks	36/109 (33.0%)	65/106 (61.3%)	< 0.0001	0.3 (0.2–0.5)
Try to get my child to finish breastmilk or formula (denominator = all available)				
16 weeks				
Never	48/132 (36.4%)	39/131 (29.8%)	0.06	0.7 (0.4–1.0)
Rarely	43/132 (32.6%)	41/131 (31.3%)		
Half of the time	23/132 (17.4%)	21/131 (16.0%)		
Most of the time	16/132 (12.1%)	22/131 (16.8%)		
Always	2/132 (1.5%)	8/131 (6.1%)		
28 weeks				
Never	46/128 (35.9%)	22/125 (17.6%)	< 0.0001	0.4 (0.2–0.6)
Rarely	54/128 (42.2%)	47/125 (37.6%)		
Half of the time	11/128 (8.6%)	26/125 (20.8%)		
Most of the time	14/128 (10.9%)	18/125 (14.4%)		
Always	3/128 (2.3%)	12/125 (9.6%)		
Try to get my child to finish his/her food (denominator = all available)				
28 weeks				
Never	42/128 (32.8%)	16/119 (13.5%)	0.0001	0.4 (0.3–0.6)
Rarely	43/128 (33.6%)	43/119 (36.1%)		
Half of the time	23/128 (18.0%)	24/119 (20.2%)		
Most of the time	17/128 (13.3%)	27/119 (22.7%)		
Always	3/128 (2.3%)	9/119 (7.6%)		
Infant Feeding Styles Questionnaire (IFSQ) – 28 weeks				
Pressure to finish	1.85 (0.61), *n* = 130	2.24 (0.74), *n* = 128	< 0.0001	0.58
Pressure to soothe	1.71 (0.62), *n* = 130	2.23 (0.82), *n* = 128	< 0.0001	0.72
Pressure with cereal	1.39 (0.65), *n* = 130	1.73 (0.90), *n* = 128	0.0003	0.43
Restrictive/amount	2.84 (1.05), *n* = 130	2.93 (1.03), *n* = 128	0.48	0.09
Restrictive/diet quality	3.93 (0.69), *n* = 130	3.87 (0.77), *n* = 128	0.51	0.08
Responsive/satiety	4.62 (0.41), *n* = 130	4.56 (0.48), *n* = 127	0.29	0.13
Structure and Control in Parenting Feeding (SCPF) – 52 weeks				
Limit exposure	4.13 (0.40), *n* = 120	4.01 (0.47), *n* = 123	0.03	0.27
Consistent feeding routines	4.18 (0.48), *n* = 120	4.00 (0.49), *n* = 123	0.006	0.37
Pressure to eat	1.78 (0.62), *n* = 120	1.99 (0.68), *n* = 123	0.01	0.32
Restriction	2.12 (0.88), *n* = 120	2.03 (0.85), *n* = 123	0.42	0.10

^a^*p* for study group difference, ANOVA or logistic regression adjusted for stratification variables

### Use of feeding to soothe (feeding as the “default” to soothe a fussy infant)

Fewer mothers in the RP group reported using feeding as the first response, immediately feeding their child when they cried at 16 weeks and 28 weeks in the absence of hunger cues (Table 5). At 8, 16, 32 and 44 weeks, mothers in the RP group reported less frequent context-based and emotion-based use of food to soothe (Table 5). At 16 weeks, RP mothers were less likely to use context-based feeding to soothe “sometimes or more often” (39/130 (30.0%) RP vs 56/123 (45.5%) control, *p* = 0.01). Fewer RP mothers reported ever using emotion-based feeding-to-soothe at 16 weeks (42/125 (33.6%) vs 59/116 (53.6%), *p* = 0.008) context-based feeding-to-soothe at 44 weeks (69/116 (59.5%) vs 94/118 (79.7%), *p* = 0.007) and emotion-based feeding-to-soothe at 44 weeks (19/115 (16.5%) vs 36/117 (30.8%), *p* = 0.01) than control. At 16 weeks, mothers in the RP group reported that beverages (breastmilk/formula and other beverages) were less effective at soothing their infant than mothers in the control group. At 32 weeks, RP mothers reported lower effectiveness of both beverages and foods to soothe their child than control. At 44 weeks, RP mothers reported lower effectiveness of beverages, but not foods than control.

**Table 5:** Effect of RP intervention on use of feeding to soothe, mean (SD)

	Parenting (*n* = 140)	Control (*n* = 139)	*p*-value^c^	Odds ratio (CI) or Cohen’s D
When infant cries, I immediately feed (denominator = all available)				
16 weeks				
Never	33/131 (25.2%)	18/129 (14.0%)	0.01	0.6 (0.4–0.9)
Seldom	57/131 (43.5%)	58/129 (45.0%)		
Half of the time	34/131 (26.0%)	37/129 (28.7%)		
Most of the time	7/131 (5.3%)	11/129 (8.5%)		
Always	0/131 (0%)	5/129 (3.9%)		
28 weeks				
Never	51/128 (39.8%)	33/126 (26.2%)	0.0009	0.5 (0.3–0.7)
Seldom	52/128 (40.6%)	46/126 (36.5%)		
Half of the time	23/128 (18.0%)	36/126 (28.6%)		
Most of the time	2/128 (1.6%)	8/126 (6.4%)		
Always	0/128 (0%)	3/126 (2.4%)		
Food to soothe: Context-based^a^				
8 weeks	2.57 (0.81), *n* = 129	2.83 (0.72), *n* = 119	0.008	0.34
16 weeks	2.50 (0.74), *n* = 130	2.76 (0.84), *n* = 123	0.009	0.33
32 weeks	2.00 (0.62), *n* = 117	2.43 (0.82), *n* = 112	< 0.0001	0.59
44 weeks	2.16 (0.70), *n* = 116	2.56 (0.76), *n* = 118	< 0.0001	0.55
Food to soothe: Emotion-based^a^				
8 weeks	1.78 (0.94), *n* = 128	2.01 (0.92), *n* = 110	0.07	0.25
16 weeks	1.57 (0.77), *n* = 125	1.90 (0.89), *n* = 116	0.002	0.40
32 weeks	1.36 (0.50), *n* = 117	1.65 (0.71), *n* = 110	0.0003	0.47
44 weeks	1.31 (0.47), *n* = 115	1.62 (0.71), *n* = 117	0.0001	0.51
Food to soothe efficacy: Beverages^b^				
8 weeks	3.09 (0.72), *n* = 129	3.31 (0.69), *n* = 122	0.02	0.31
16 weeks	2.93 (0.76), *n* = 123	3.15 (0.68), *n* = 128	0.01	0.31
32 weeks	2.55 (0.77), *n* = 100	2.77 (0.75), *n* = 104	0.03	0.29
44 weeks	2.36 (0.70), *n* = 97	2.56 (0.75), *n* = 104	0.046	0.28
Food to soothe efficacy: Solid Foods^2^				
32 weeks	1.83 (0.48), *n* = 46	2.06 (0.45), *n* = 54	0.02	0.49
44 weeks	2.03 (0.46), *n* = 58	2.11 (0.57), *n* = 73	0.43	0.15

Notes. ^a^Scale ranged from 1 (never) to 5 (always) ^b^Scale ranged from 1 (does not work) to 4 (works all of the time)

^c^*p* for study group difference, ANOVA or logistic regression adjusted for stratification variables

### Food intake

At 1 year, there were few differences between groups in dietary intake as measured by the FFQ. Fewer mothers in the RP group reported that their child consumed salty snacks (chips, crackers, pretzels, etc.) on a daily basis compared to the control group (12/122 (9.8%) vs 24/118 (20.3%), *p* = 0.03), and more mothers in the RP group reported that their child consumed vegetables daily (117/122 (95.9%) vs 105/118 (89.0%), *p* = 0.049), but there were no study group differences in reported daily or weekly exposure to fruit juice, sugar sweetened-beverages, sweets, fried foods, or fruit. Mothers who were predominantly breastfeeding at 16 weeks were less likely to report that their child consumed any sugar sweetened beverages (6/120 (5.0%) vs 21/119 (17.7%), *p* = 0.02) or fruit juice (46/122 (37.7%) vs. 74/124 (59.7%), *p* = 0.009) in the past week at 1 year than those that formula feeding at 16 weeks, but there was no interaction between predominant milk-feeding type and study group (data not shown). There were no other significant differences in daily or weekly exposure to food groups by predominant milk feeding type.

## Discussion

Results provide support that the RP intervention influenced how first-time mothers were feeding their infants, and to a lesser extent *what* infants were fed. The RP mothers reported both less frequent use of non-responsive feeding practices that could promote excessive intake (i.e., feeding to soothe, pressuring to finish a bottle) and more frequent use of structure-based feeding (i.e., limit-setting, consistent feeding routines). Compared to control mothers, RP mothers also reported that feeding was less effective at soothing their infant. This may be because mothers were using alternatives to using food to soothe (e.g., swaddling swinging, pacifier), instead of using food as a first response to crying. Combined with evidence of the RP intervention’s effects on infant sleep health and dietary intake patterns [32, 33], data from this study contribute to identification of modifiable parenting behaviors associated with less rapid infant weight gain from birth to 28 weeks and lowered risk of overweight at 1 y as previously described [26]. INSIGHT was designed as a multicomponent RP intervention based on the perspective that infant feeding is a key aspect of early caregiving related to infant arousal (i.e., fussing and crying), sleep, and active social play. Although our RP approach is novel with respect to early obesity prevention, these findings provide one more piece of evidence that RP guidance can promote beneficial parenting and child outcomes [15–18].

INSIGHT RP guidance focused on feeding in response to infant hunger and fullness, and on alternative soothing strategies to calm a non-hungry, distressed infant. Early in infancy, when exclusively milk-fed, infants are learning which cues are associated with initiation and termination of eating [34]. Infants need to learn to discriminate hunger from other distress, requiring sensitive parenting. In other words, parents should feed in response to hunger, but use other non-food approaches with distress that is not hunger-related (e.g., tired, scared, overstimulated). Repeated use of feeding to soothe a distressed, non-hungry infant pairs feeding with removal of non-hunger distress cues has been shown to influence infant weight [8, 24, 35]. Although the mechanism is not clear, preschool children whose mothers report greater use of food to regulate their infant’s emotions have infants who are more likely to develop emotional eating styles and poorer diets [36, 37].

In this study, relative to the control, RP mothers reported that feeding was a less effective way to soothe their infant, which could have persistent effects on how mothers use food to manage child behavior as the child develops [38]. Using food to soothe infant distress could result in conditioned eating in response to negative emotions, a characteristic of emotional eaters that is associated with obesity in older children [39, 40]. More evidence is needed, but is it possible that children are learning to eat in response to emotional cues. Pressuring the infant to finish the bottle can also increase infant intake and perhaps affect the extent to which infants attend to or ignore fullness cues in determining meal termination. Over time this can affect learning by shaping the extent to which internal fullness cues and amount of food remaining affect amount consumed, and ability to self-regulate. Serving children larger portions promotes increased intake [41] and other experimental research with young children has shown that pressuring children to eat in order to finish consuming food on their plate can promote greater intake [38, 42–45].

RP intervention parents were less likely to report that they used feeding as the immediate, default response to infant crying and reported less frequent use of feeding to soothe. Compared to control, mothers in the RP group reported that both milk and solid foods were less effective at soothing their infant. This may because RP mothers had learned to use alternative approaches to soothing the infant, or they paid closer attention to their infant’s signals, using soothing techniques to calm a distressed infant, although direct evidence on this point is lacking. Infants cry for many reasons, hunger being just one. A potential explanation is that the RP group may just be less dependent on using food for non-nutritive soothing. Together, findings from this study provide support that an early parenting intervention can affect *how* and in what contexts infants are being fed, at least among first-time parents, and that how infants are fed can impact growth and weight outcomes.

Although The INSIGHT RP curriculum also targeted *what* foods infants consumed by providing traditional nutrition education to parents on age-appropriate foods and portions, results indicate that the RP intervention had very limited effects on what drinks and foods were consumed; the INSIGHT RP intervention did not affect the extent to which infants were offered fruit juice, sugar sweetened beverages, sweet foods, or fried foods at age 1 year. We previously reported differences in dietary *patterns* at 9 months between RP and control, particularly among formula-fed infants [33]. However, it is plausible that by age 1 year, most infants had nearly completed the transition to consuming diets very similar to those of other family members. Given this and the few observed group differences on diet quality, novel family-based interventions targeting the home food and eating environment may be needed to impact the family diet during this critical developmental period when the infant is transitioning to the adult diet. It is unlikely that interventions can impact “what” foods infants and toddlers are exposed to without also changing what their parents are eating.

A study limitation is that all feeding behavior and food intake data were self-reported; RP mothers may have provided socially desirable responses. However, differences between treatment groups were much less evident for what was fed (e.g., sugar sweetened beverages, sweet and salty foods, fruits and vegetables), despite the fact that it is common knowledge that some foods are considered to be healthy for children (e.g., vegetables) and others are not (e.g., SSBs, fried foods). Mothers may have systematically under-reported the “unhealthy” foods. In addition to INSIGHT guidance on feeding, parents receive messages about what to eat and what not to eat from many other sources, including their pediatricians, the Special Supplemental Nutrition program for Women, Infants, and Children (WIC), in addition to pervasive marketing. This provides additional evidence for including 0–2 years in the Dietary Guidelines. Given the INSIGHT findings and national data, novel interventions targeting the family eating environment may be needed to impact the family diet when the infant is transitioning to the adult diet. Lastly, our sample was relatively demographically homogenous and is not nationally representative, limiting the generalizability of our findings.

## Conclusions

The INSIGHT RP intervention affected maternal feeding practices. During the first year, RP mothers were consistently less likely to report using feeding to soothe their infant for reasons other than hunger, and less likely to report that feeding was an effective strategy for soothing their infant. Because RP mothers learned that alternative soothing strategies could be effective in soothing their infant, they may have been less likely to feed in response to contextual or emotional cues. RP mothers were also encouraged to terminate feedings in response to infant cues. RP mothers were less likely to report trying to get their infant to finish a bottle or other food. In contrast, RP mothers were more likely to use structure and routines in feeding. Taken together, these early differences in maternal feeding suggest that RP and control infants may have had different early experiences with feeding; in particular, the extent to which environmental or internal cues predicted the onset and termination of feedings, affecting the extent to which infants eat in response to hunger or in response to other distress. Findings support the view that providing anticipatory guidance on RP in feeding can promote use of more sensitive, structure-based feeding, which could reduce obesity risk by affecting how and when infants are fed. These findings, in combination with others from INSIGHT indicate that the RP intervention can affect multiple outcomes including feeding, sleeping [32] and early growth and obesity risk in infancy [26].

## Additional file

Additional file 1: **Appendix 1.** Factor loadings for questionnaire items indicating contextual and emotional food-to-soothe feeding practices. Exploratory factor analysis was completed using unweighted least squares with an equamax rotation, restricting to 2 factors. (N=197). (PDF 500 kb)

## Abbreviations

BMI: Body Mass Index
INSIGHT: Intervention Nurses Start Infants Growing on Healthy Trajectories
RP: Responsive Parenting
