# Supplementary material for: INSIGHT responsive parenting intervention and infant feeding practices: randomized clinical trial
Source: Int J Behav Nutr Phys Act. 2018 Jul 9;15:64. doi: 10.1186/s12966-018-0700-6 (PMC6038199; doi:10.1186/s12966-018-0700-6)
Supplement: Supplementary file 1 — Appendix 1. Factor loadings for questionnaire items indicating contextual and emotional food-to-soothe feeding practices. Exploratory factor analysis was completed using unweighted least squares with an equamax rotation, restricting to 2 factors. (N=197). (PDF 500 kb) [file 12966_2018_700_MOESM1_ESM.pdf]

Appendix 1. Factor loadings for questionnaire items indicating contextual and emotional food-to-soothe feeding practices. Exploratory factor analysis was completed using unweighted least squares with an equamax rotation, restricting to 2 factors. (N=197).

| Question: How likely are you to use food to soothe when . . . | | Factor 1: emotional | Factor 2: context |
|---|---|---|---|
| | You are in a doctor's waiting room?[2] | 0.09 | **0.74**[1] |
| | You are shopping in a store? | 0.19 | **0.65**[1] |
| | You are in the car? | 0.20 | **0.54**[1] |
| | You are getting ready to leave the house? | 0.29 | **0.65**[1] |
| | Before naptime or bedtime? | 0.08 | **0.44**[1] |
| | How likely to use food to soothe when nothing else works? | 0.32 | **0.48**[1] |
| | You are stressed? | **0.92**[1] | 0.17 |
| | You are frustrated/upset? | **0.90**[1] | 0.15 |
| | You are tired? | **0.81**[1] | 0.21 |
| | You are busy? | **0.71**[1] | 0.32 |
| | Your child wants attention? | **0.64**[1] | 0.22 |
| | Your child is frustrated/angry? | **0.54**[1] | 0.35 |
| | | | |
| Items that did not load | | | |
| | In church or other place of worship | | |
| | When you are preparing meals? | | |

| | | | |
|---|---|---|---|
| | | | |
| | When you are on the phone? | | |
| | When your child wakes during the night? | | |
| | To occupy your child until meal time? | | |
| | While doing household chores? | | |
| | When your child is scared? | | |
| | When your child is left alone or with unfamiliar people? | | |
| | When your child is in their car seat or otherwise has their movement restricted | | |
| | [1] Factor loadings that contribute to defining each factor.<br>[2] Responses are scored on a 5-point likert scale: 1 = never to 5 = always. | | |
